# 3D Printable Composite Biomaterials Based on GelMA and Hydroxyapatite Powders Doped with Cerium Ions for Bone Tissue Regeneration

**DOI:** 10.3390/ijms23031841

**Published:** 2022-02-06

**Authors:** Rebeca Leu Alexa, Andreia Cucuruz, Cristina-Daniela Ghițulică, Georgeta Voicu, Liliana-Roxana Stamat (Balahura), Sorina Dinescu, George Mihail Vlasceanu, Cristina Stavarache, Raluca Ianchis, Horia Iovu, Marieta Costache

**Affiliations:** 1 Advanced Polymer Materials Group, Department of Bioresources and Polymer Science, University Politehnica of Bucharest, Gheorghe Polizu 1-7, 011061 Bucharest, Romania; leurebeca@gmail.com (R.L.A.); crisstavarache@gmail.com (C.S.); horia.iovu@upb.ro (H.I.); 2 Department of Biomaterials and Medical Devices, Faculty of Medical Engineering, University Politehnica of Bucharest, Gheorghe Polizu 1-7, 011061 Bucharest, Romania; vlasceanu.georgemihail@yahoo.ro; 3 Department of Science and Engineering of Oxide Materials and Nanomaterials, Faculty of Applied Chemistry and Materials Science, University Politehnica of Bucharest, Gheorghe Polizu 1-7, 011061 Bucharest, Romania; cristina.ghitulica@upb.ro (C.-D.G.); georgeta.voicu@upb.ro (G.V.); 4 Department of Biochemistry and Molecular Biology, Faculty of Biology, University of Bucharest, 050095 Bucharest, Romania; roxana.balahura@bio.unibuc.ro (L.-R.S.); marieta.costache@bio.unibuc.ro (M.C.); 5 Research Institute of the University of Bucharest, 050663 Bucharest, Romania; 6 Costin D. Nenitescu, Centre of Organic Chemistry, 202-B Spl. Independentei, 060023 Bucharest, Romania; 7 National Institute for Research & Development for Chemistry and Petrochemistry ICECHIM—Bucharest, Spl. Independentei 202, 6th District, P.O. Box 35/174, 060021 Bucharest, Romania; ralumoc@yahoo.com; 8 Academy of Romanian Scientists, Splaiul Independentei 54, 050094 Bucharest, Romania

**Keywords:** 3D printing, hydroxyapatite doped, cerium, gelatin methacryloyl, biological analyses

## Abstract

The main objective was to produce 3D printable hydrogels based on GelMA and hydroxyapatite doped with cerium ions with potential application in bone regeneration. The first part of the study regards the substitution of Ca^2+^ ions from hydroxyapatite structure with cerium ions (Ca_10-x_Ce_x_(PO_4_)_6_(OH)_2_, xCe = 0.1, 0.3, 0.5). The second part followed the selection of the optimal concentration of HAp doped, which will ensure GelMA-based scaffolds with good biocompatibility, viability and cell proliferation. The third part aimed to select the optimal concentrations of GelMA for the 3D printing process (20%, 30% and 35%). In vitro biological assessment presented the highest level of cell viability and proliferation potency of GelMA-HC5 composites, along with a low cytotoxic potential, highlighting the beneficial effects of cerium on cell growth, also supported by Live/Dead results. According to the 3D printing experiments, the 30% GelMA enriched with HC5 was able to generate 3D scaffolds with high structural integrity and homogeneity, showing the highest suitability for the 3D printing process. The osteogenic differentiation experiments confirmed the ability of 30% GelMA-3% HC5 scaffold to support and efficiently maintain the osteogenesis process. Based on the results, 30% GelMA-3% HC5 3D printed scaffolds could be considered as biomaterials with suitable characteristics for application in bone tissue engineering.

## 1. Introduction

Producing novel scaffold materials to replace or repair bone defects offers great potential when considering the 2.2 million bone graft surgeries performed worldwide annually [1,2,3]. Currently, research in the field of tissue engineering and implicitly that of regenerative medicine, focuses on the development of biomaterials that restore, maintain or improve tissue function, materials that could revolutionize bone therapy surgery for millions of patients. Tissue engineering aims to obtain bone grafts with regeneration potential both in vitro and in vivo. These types of materials are usually bioactive and have the ability to repair bones through guided tissue regeneration [4,5,6,7]. Some of the most common biomaterials used for such applications are calcium phosphates, especially hydroxyapatite.

Calcium phosphate is the most widely used and studied material because of its great compatibility and similarity in terms of chemical composition with the mineral phase founded in hard tissues [8,9]. Thus, to better mimic the composition of natural bone, literature studies suggest that hydroxyapatite should be doped with various divalent, trivalent or tetravalent ions, such as Sr^2+^, Mg^2+^, Zn^2+^, Ce^3+^, La^3+^, Ce^4+^. These substitutions were demonstrated to improve the biological and mechanical properties of hydroxyapatite. The first part of the present study regards the substitution of Ca^2+^ ions from hydroxyapatite structure with cerium ions, which have an ionic radius very close to that of calcium—Ce^3+^ (1.04 Å) and Ce^4+^ (0.97 Å) compared to Ca^2+^ (1.06 Å). Moreover, the substitution with cerium ions improves the chemical and biological properties of calcium phosphates but can also have an antibacterial action inhibiting the development of pathogens (Escherichia coli, Pseudomonas aeruginosa, Staphylococcus aureus and Bacillus subtilis). Several research studies also suggested that cerium ions had a positive effect on the processes of proliferation, differentiation and mineralization of osteoblasts [10,11].

On the other hand, 3D printing is one of the most employed techniques to generate 3D scaffolds able to support cells for tissue engineering and regenerative medicine. Soft materials 3D printing allows the use of hydrogel-based materials with proper viscosity to design various 3D architectures, which could also serve as a proper medium for cell encapsulation or cell seeding [12].

In this respect, a multitude of hydrogel-based bio-inks have been studied, such as those obtained from gelatin, alginate, chitosan, fibrinogen and so on [13,14,15].

In order to be used in 3D printing, hydrogels must present suitable viscosity to preserve the architecture of scaffolds during and after the printing process. Moreover, the hydrogel-based biomaterials must present shear thinning behavior, biocompatibility, biodegradability and the capacity to assure cell attachment and proliferation.

Gelatin represents one of the most studied proteins with regard to obtaining 3D printable hydrogels because of its characteristics, such as high biocompatibility, biodegradability, water solubility, nontoxicity and nonimmunogenicity as well as the ability to mimic the natural extracellular matrix (ECM). Moreover, owing to the target sequences for the enzymatic degradation of matrix metalloproteinases (MMPs), gelatin also presents bioresorbability and its degradation products are biocompatible. Gelatin can be considered a biomimetic peptide as it promotes cell adhesion, prevents apoptosis and accelerates tissue regeneration [16,17].

Additionally, gelatin is a biomass resource [18], presenting low cost and ease of manipulation.

However, the disadvantages of gelatin-based biomaterials are mainly linked to the weak mechanical properties and accelerated degradation in physiological medium [17,19,20]. Still, gelatin presents a folding structure that can be easily modified, with these drawbacks being eliminated by simple crosslinking. In our study, gelatin was modified by methacrylic anhydride to obtain methacrylate groups that can be easily crosslinked by photopolymerization (UV, 365 nm) at room temperature, thus providing good temporal control over the crosslinking process without compromising the cell viability [17,21].

Gelatin methacryloyl (GelMA) has recently gained increasing attention in the production of 3D printable hydrogels because of its biocompatibility and ability to be photocrosslinked [22]. Additionally, GelMA exhibit cell-responsive characteristics, such as promoting cell adhesion, proliferation and differentiation [23].

Irgacure 2959 was selected as a photoinitiator for the crosslinking process because of its good solubility in water, low cytotoxicity compared to other photoinitiators and besides this, it does not affect cell viability [21].

GelMA photocrosslinking has another great advantage, namely, it can be employed in different additive manufacturing techniques, such as extrusion, stereolitography, digital light processing (DLP), high resolution laser-based bioprinting and volumetric bioprinting, and is useful in diverse applications such as bone, dermal, cartilage, neural or cornea tissues regeneration [24].

Instead, in order to effectively control the GelMA viscosity, Choi et al. proposed a two-stage temperature control system and GelMA composite scaffolds were fabricated using silanated silica particles of different concentrations (0, 3 and 10 wt%) via extrusion-based 3D printing technique. GelMA-based composite scaffolds indicated potential for application in hard tissue regeneration as the composite scaffold exerted improved physical and biological performances [25].

Other studies involved clay nanoparticles as inorganic reinforcers for printing stable 3D GelMA-based composite constructs by extrusion technique also for hard tissue regeneration. Thus, by varying the amount but also the type of nanoclay included, the viscosity of the printing ink can be tailored, as can the porosity, mechanical strength and degradation ratio of the printed scaffolds, while maintaining a good biocompatibility [16,26].

Another study followed the 3D printing of suitable cell friendly GelMA hydrogel and mechanically strong biodegradable polymer PCL into a multilayered scaffold through a multi-head deposition method. The obtained 3D hybrid constructs demonstrated good viability, osteogenic differentiation and mineralization but also, suitable mechanical features for proper bone tissue engineering applications [27].

Gelatin methacrylate and hydroxyapatite were used in the additive manufacturing of a trilayered scaffold via extrusion-based multi nozzle 3D printing following UV crosslinking in the presence of lithium phenyl-2,4,6-trimenthylbenzoylphosphinate (LAP) photoinitiator. These complex scaffolds were successfully evaluated for in vivo repair of rabbit osteochondral defects that were envisaged for patient specific cartilage and bone regeneration [28].

The main target of this study was to obtain 3D printable hydrogels based on GelMA and hydroxyapatite doped with cerium ions with potential application in bone regeneration. The first part of the study regards the substitution of Ca^2+^ ions from hydroxyapatite structure with cerium ions. The second part followed the selection of the optimal concentration of HAp doped, which will ensure GelMA-based scaffolds with good biocompatibility, viability and cell proliferation. The third part aimed to select the optimal concentrations of GelMA for the 3D printing process.

## 2. Results and Discussion

### 2.1. Characterization of Ceramic Powders, GelMA and Composite Materials

#### 2.1.1. X-ray Diffraction (XRD)

**Figure 1:**
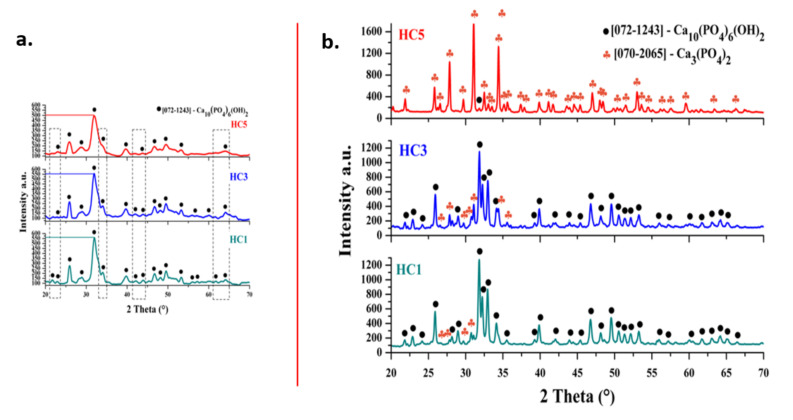
XRD patterns of: (**a**) Dry precipitates at 80 °C for HC1, HC3 and HC5; and (**b**) Calcinate ceramic powders at 900 °C for HC1, HC3 and HC5.

Figure 1a illustrates the phase composition and lattice parameter for the dry precipitates of samples HC1, HC3 and HC5. All samples exhibited the typical hexagonal crystalline behavior of HAp (space group P63/m) with corresponding peaks of 2 theta at 21.7° (2 0 0), 22.8° (1 1 1), 25.8° (0 0 2), 28.8° (2 1 0), 31,7° (2 1 1), 34° (2 0 2), 39.7° (1 3 0), 41.9° (1 3 1), 43.8° (1 1 3), 45.2° (2 0 3), 46.6° (2 2 2), 48° (1 3 2), 49.4° (2 1 3), 53,1° (0 0 4), 55.8° (3 2 2), 57° (3 1 3) and 64.1° (3 2 3) (JCPDS No. 072-1243). In general, the spectra collected from the dry precipitates HC1, HC3 and HC5 are similar. However, the addition of cerium ions in the HAp structure makes the diffraction peaks slightly wider; moreover, it has been observed that the relative intensity of all diffraction peaks slightly decreases as the amount of cerium in the HAp structure increases. Literature studies [29,30] suggest that these aspects may be due to the mechanism of substitution of Ca^2+^ ions with Ce^3+^ or Ce^4+^. This indicates that solid HAp solutions with Ce^3+^ or Ce^4+^ incorporated in its structure were formed in the composition. This network substitution occurs easily, and this may be due to similar ionic rays, Ce^3+^ (1.04 Å) and Ce^4+^ (0.97 Å) compared to Ca^2+^ (1.06 Å).

Figure 1b shows the diffraction spectra of HC1, HC3 and HC5 ceramic powders calcined at 900 °C. All three types of samples exhibited a crystalline structure, in which the hexagonal crystalline structure of HAp, similar to dry precipitates (JCPDS No. 072-1243), is identified as a phase. This time, the hexagonal crystal structure of HAp is not the only phase, all three samples subjected to heat treatment, have another crystalline phase—tricalcium phosphate with a rhombohedral structure (space group R3c) (β-TCP) and wherein its proportion increases with increasing cerium content of the samples (HC1 → HC3 → HC5) (JCPDS No. 070-2065). The characteristic peaks of the rhombohedral crystal structure of TCP at 2 θ are 26.4° (1 2 2), 29.6° (3 0 0) and 31° (0 2 10) for the HC1 sample, 26.4° (1 2 2), 27.7° (2 1 4), 29.6° (3 0 0), 30.9° (0 2 10), 34.3° (2 2 0) and 35.5° (2 1 10) for the HC3 sample and 25.8° (1 0 10), 27.7° (2 1 4), 30.9° (0 2 10) and 34.3° (2 2 0) for the HC5 sample. Additionally, for all three samples, the average diameter of crystallite, D (nm) was calculated using the Debye–Scherrer equation, resulting in a value of 33.64 nm for HC1 powder, 38.69 nm for HC3 powder and 50.06 nm for HC5 powder.

#### 2.1.2. Laser Granulometry

**Figure 2:**
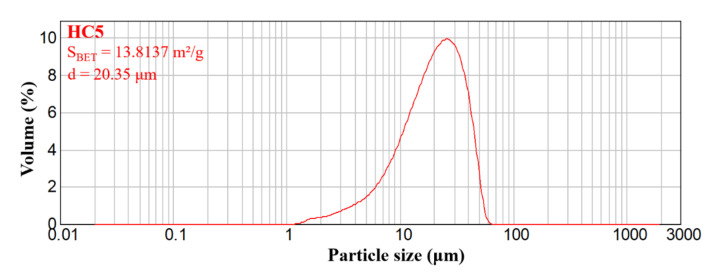
Grain size distribution for the ceramic powder.

The powder obtained after the calcination heat treatment was characterized in terms of dispersion, by determining the specific BET surface and the particle size distribution by laser granulometry. Figure 2 presents the monomodal particle size distribution of the powder, with an average particle size of 20.35 μm and a specific BET area of approximately 13.81 m^2^/g; these data show that the powder is characterized by a high fineness.

#### 2.1.3. ^1^H-NMR Spectrometry

Grafting of the methacrylate groups on the gelatin backbone took place by the reactions of residues units of arginine and methacrylic anhydride. Through ^1^H-NMR spectrometry, the functionalization degree of gelatin with methacrylate groups could be determined.

**Figure 3:**
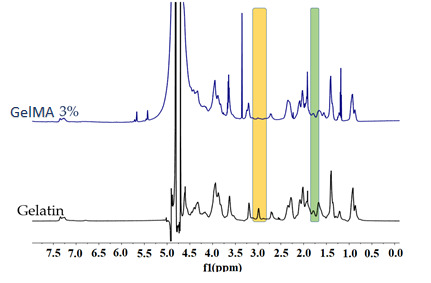
1H-NMR spectra of gelatin and GelMA.

The new signals from δ = 5.4 and 5.7 ppm that correspond to the acrylic protons of methacrylic functions from the methacrylic anhydride structure validate the synthesis of GelMA. Additionally, the synthesis of GelMA was confirmed by the methyl group signal at 1.9 ppm that increased and by decrease in the arginine signal at 2.85 ppm (Figure 3) [17]. Methacrylation degree of GelMA with 3% methacrylic anhydride was 79%.

#### 2.1.4. Fourier Transform Infrared Spectrometry—FTIR

**Figure 4:**
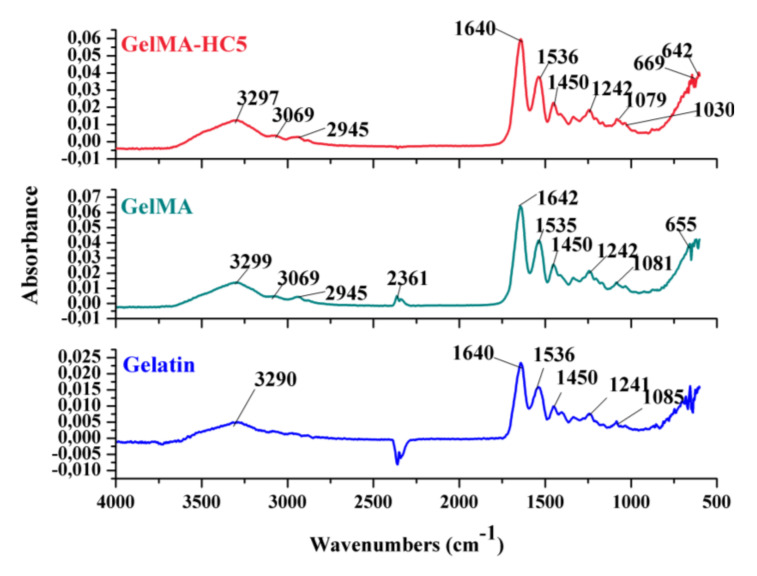
FT-IR spectra for Gelatin, GelMA and GelMA-HC5 composites.

The FTIR spectra registered for methacrylate gelatin and HC5 composites are presented in Figure 4.

The FTIR spectrum of gelatin shows a wide absorption band at 3200–3400 cm^−1^, which can be attributed to the tensile vibration of the NH bond in amide A. This overlaps with the signal recorded by the tensile vibration of the O-H bonds in the structure of the hydroxyproline amino acid, which is found in the composition of gelatin. Furthermore, the signal from 3069 cm^−1^ is attributed to the tensile vibration of the C-H bond in amide B [5,9] and three signals characteristic of the functional groups in the primary structure of proteins are highlighted: the absorption band from 1640 cm^−1^ specific to the vibration of the C=O bond of amide I, the maximum absorption from 1536 cm^−1^ specific to the tensile and deformation vibrations C-N and N-H of amide II, respectively, the signal from 1241 cm^−1^ attributed to the bond deformation vibration N-H specific for amide III in the structure of gelatin, according to data from the literature [31,32].

The absorption bands characteristic of the functional groups in the polymer structure are also found in the FTIR spectrum recorded for GelMA, which are positioned at the same wave number. This indicates that the peptide bonds (-NH-CO-) between the amino acids in the primary structure of the protein were not affected in the methacrylation step.

The FTIR spectra recorded for composite materials (GelMA and cerium-doped hydroxyapatite) indicated that the characteristic peaks of the mineral phase are superimposed with those of the gelatin, by the presence of the phosphate (PO_4_^3^^−^) and hydroxyl (OH^−^) groups. Furthermore, in addition to the bands discussed above, the wide peak at 3297 cm^−1^ can be attributed to OH^−^ stretching vibrations, and the peaks at 1079 cm^−1^ and 1030 cm^−1^ are assigned to PO_4_^3^^−^ υ_3_ and PO_4_^3^^−^ υ_2_ [8,9,33].

#### 2.1.5. Scanning Electron Microscopy

**Figure 5:**
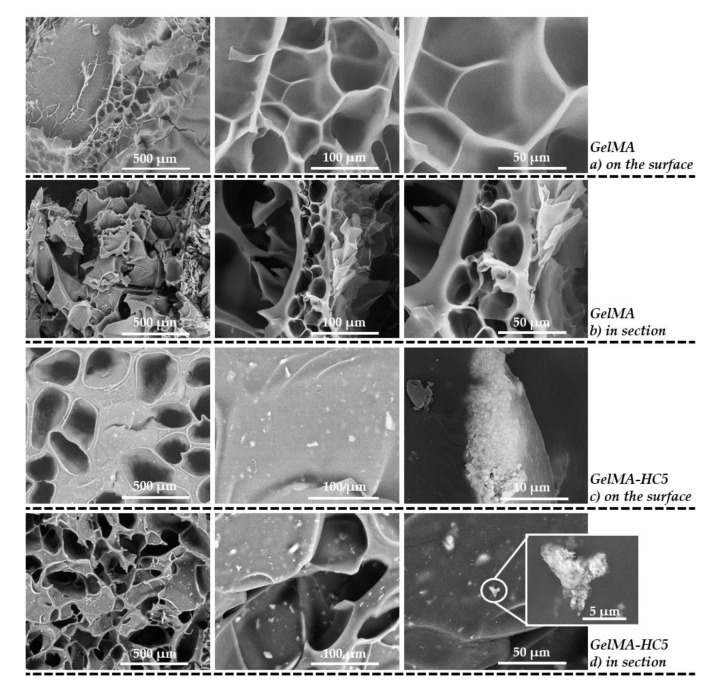
SEM micrographs recorded on GelMA samples: (**a**) on the surface, and (**b**) in section. GelMA-HC5 samples: (**c**) on the surface, and (**d**) in section.

SEM micrographs were recorded on the control sample-GelMA (Figure 5a,b). Scanning electron microscopy images evidenced a porous microstructure of methacrylate gelatin, with evenly distributed and interconnected pores. According to literature studies, the freeze-drying procedure influences the structure of the material and the porosity degree of this microstructure.

Therefore, the material exhibits adequate structural characteristics for the incorporation of the mineral phase-hydroxyapatite doped with cerium ions. The interconnected pores are beneficial to incorporate higher cell density, cells migration and differentiation.

SEM micrographs from Figure 5c,d are those obtained for GelMA-HC5 composites, which present a porous microstructure. The pores are interconnected and evenly distributed with different sizes. The presence of fine particles on the surface of the material is noticed, and at larger magnifications, it can be observed that these particles are present in the mass of the material. This is due to the fact that the ceramic powder is embedded in the matrix of methacrylate gelatin, given that obtaining the composite materials took place by homogenizing the hydroxyapatite powder in a polymeric solution, which was photopolymerized and subsequently lyophilized. The particles in the form of aggregates have sizes in the range of 150–180 nm.

For both the methacrylate gelatin sample and the GelMA-HC5 composites, the internal porous microarchitecture of the sections is evidenced in Figure 5. Methacrylate gelatin has an average pore length of 31.17 μm and an average pore width of 23.51 μm, while composite samples of methacrylate gelatin in which the mineral phase of hydroxyapatite doped with cerium ions was incorporated, has an average pore length of 254.36 μm and an average pore width of 134.37 μm. It is possible that the mineral phase influences the morphology of the pores.

**Figure 6:**
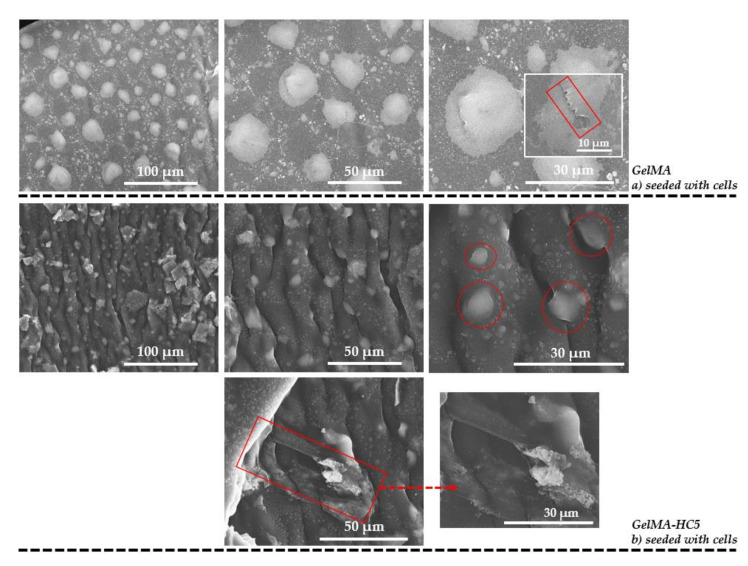
SEM micrographs recorded on: (**a**) GelMA, and (**b**) GelMA-HC5 composites seeded with cells.

Additionally, methacrylate gelatin-GelMA and GelMA-HC5 composites—were seeded with a density of 2 × 10^5^cells/cm^2^ type murine preosteoblasts from MC3T3-E1 cell line. SEM micrographs are presented in Figure 6. The presence of uniformly attached cells on the surface of the samples was identified on both types of samples. Furthermore, in the case of GelMA-HC5 composite materials, it can be seen how some of these cells anchored to the surface of the material. According to some research studies [18], the cells form filopodia that allow this surface anchoring and subsequently a good spread. These types of interactions have direct effects on the spatial organization of the cytoskeleton, and another important aspect is the surface roughness of the material, for good cell adhesion.

#### 2.1.6. Biological Analyses

- Cell viability

**Figure 7:**
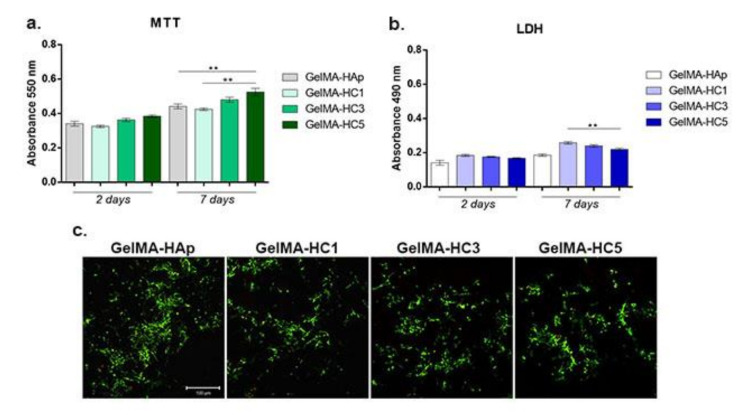
(**a**) Cell viability and proliferation profile evaluation by MTT assay at two and seven days after cell seeding. Statistical significance: ** *p* < 0.01. (**b**) GelMA-HAp enriched scaffolds cytotoxicity evaluation by LDH assay at two and seven days after cell seeding. Statistical significance: ** *p* < 0.01. (**c**) Fluorescence microscopy evaluation of living cells (green) and dead cells (red) in contact with GelMA-HAp enriched scaffolds.

Cell viability and proliferation profile assessment by MTT assay revealed an overall beneficial interaction between cells and GelMA-HAp enriched scaffolds after two and seven days of culture in standard conditions (Figure 7a). After two days of culture in standard conditions, slight differences in cell viability profiles were registered in contact with HAp-Cerium derived scaffolds. After seven days of culture, statistical significant differences were observed between the GelMA-HAp composite and GelMA-HC5 (*p* < 0.01) suggesting beneficial effects of cerium on cell growth. Additionally, the highest level of cell viability and proliferation were observed for GelMA-HC5 composite, in comparison with GelMA-HC1 composite (*p* < 0.01). Previous studies [10] investigated the effect of doped-hydroxyapatite containing 5% (w/w%) cerium, cashew gum and gellan gum in contact with rabbit mesenchymal stem cells. The results showed that the proposed scaffold supported cell viability and cell growth, that are suitable for bone regeneration applications.

- Materials cytotoxicity

LDH assay revealed the cytotoxic potential of the GelMA-HAp enriched scaffolds in contact with murine preosteoblasts. Relatively low levels of LDH were detected for all composites, showing an overall good biocompatibility of the tested scaffolds (Figure 7b). After two days of culture, no significant differences were registered in terms of cytotoxicity between various concentrations of cerium added into the scaffolds’ composition. In contrast, after seven days of culture, a significant increase was registered in the amount of dead cells in contact with GelMA-HC1 compared to the level found for GelMA-HC5 (*p* < 0.01).

- Live/Dead assay

Murine preosteoblasts were evaluated for viability and proliferation in direct contact with the GelMA-HAp scaffolds enriched with HC1, HC3, HC5 compared to the cells cultured on simple GelMA-HAp control scaffold (Figure 7c). Fluorescent Live/Dead staining and confocal microscopy allowed the qualitative evaluation of cell distribution and viability in contact with GelMA-HAp enriched composites. Live/Dead fluorescence microscopy results are in accordance with the quantitative results obtained from MTT and LDH assays.

A positive ratio between live cells and dead cells was identified in all composites, confirming the biocompatibility of all tested biomaterials. The highest cell density was identified in contact with GelMA-HC5, although no major differences were noticed between the HAp-Cerium derived scaffolds ability to support cell viability. All scaffolds displayed biocompatibility, with a much higher proportion of living cells than dead cells.

### 2.2. Characterization of Scaffolds Obtained from Nanocomposite Hydrogel Printing Inks—20%GelMA-3%HC5, 30%GelMA-3%HC5, 35%GelMA-3%HC5

#### 2.2.1. Printability

Because the main goal of this study was to obtain 3D printable bioinks that can be used in bone regeneration, the following biology results showed that GelMA and HAPC5 hydrogels presented the best biological properties, such as biocompatibility and biodegradability qualities, mimicking the complexity of native tissue by promoting cell proliferation, three nanocomposite hydrogels based on different concentrations of GelMA and constant HC5 were developed and subjected to printing tests.

**Figure 8:**
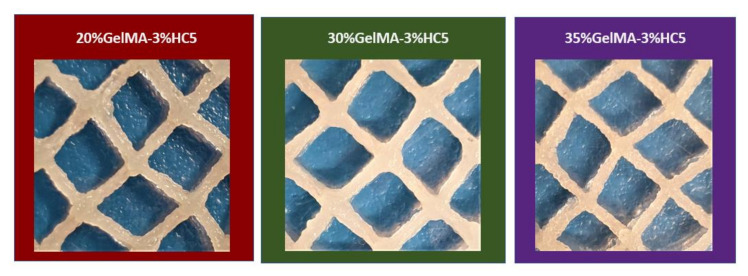
3D printed scaffolds based on nanocomposite hydrogels 20% GelMA-3% HC5, 30% GelMA-3% HC5, 35% GelMA-3% HC5.

All samples were printed at room temperature (Figure 8).

Firstly, the printing ink based on 20% GelMA-3% HC5 and 1% Irgacure was subjected to 3D printing. A 23G needle, 120 kPa pressure and 8 mm/s print speed were used. After the printing process, the obtained layered 3D scaffolds were photo crosslinked under UV light (365 nm). Filaments started to collapse after deposition of 10 layers due the low stability of the material.

In order to improve the apparent viscosity, the concentration of GelMA was increased at 30% and 35%. For both hydrogels, 25G needles, pressures of 160 kPa for 30% GelMA, respectively, 190 kPa for 35% GelMA and printing speeds of 4 mm/s, were settled. In this case, scaffolds maintained their structural integrity till 20 layers, and the filaments spread less than for 20% GelMA-3% HC5 when the material came into contact with the blades surface. Unlike the 3D printed scaffold based on 30% GelMA-3% HC5 Cerium, which presented homogeneous filaments, the scaffold obtained based on 35% GelMA-3% HC5 presented wrinkled filaments due to the high concentration of GelMA used.

Following the obtained results, the 3D printable hydrogel based on 30% GelMA-3% HC5 presented the optimal characteristics, such as structural integrity and homogeneity, to be used in the 3D printing process.

#### 2.2.2. Micro-CT

**Figure 9:**
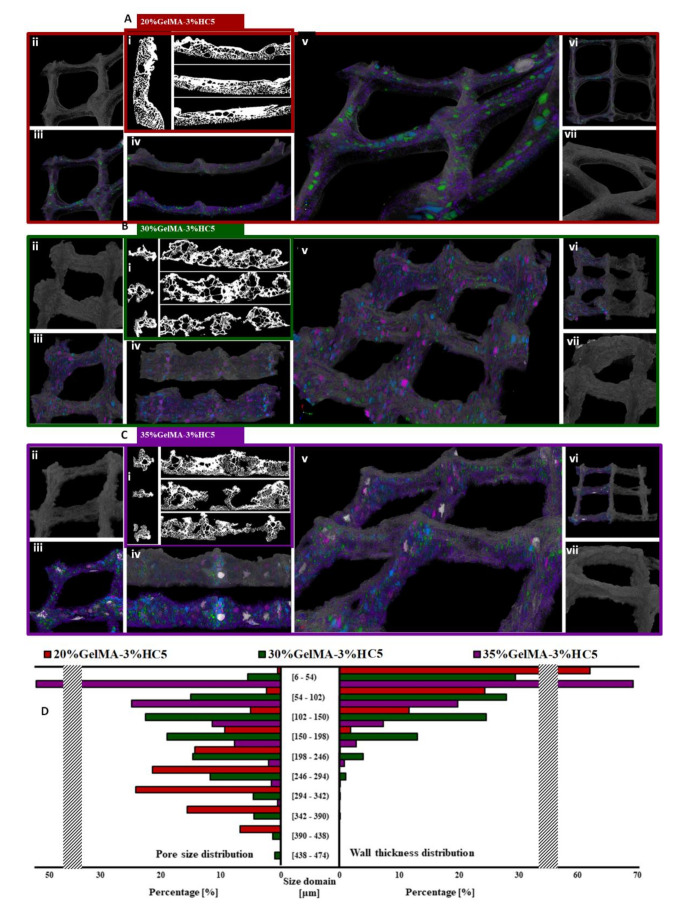
Micro-CT images of: (**A**) 20%GelMA-3%HC5, (**B**) 30%GelMA-3%HC5, (**C**) 35%GelMA-3%HC5 3D printed composites, and (**D**) Depicts the pore size and wall thickness distributions within the aforementioned specimens.

Figure 9 Micro-CT images of Figure 9A—20% GelMA-3% HC5, Figure 9B—30% GelMA-3% HC5, Figure 9C—35% GelMA-3% HC5 3D printed composites. Subsection Figure 9D depicts the pore size and wall thickness distributions within the aforementioned specimens; plotted values resulted from the 3D analysis performed in CTAn for the whole sample dataset. In Figure 9A–C, Figure 9(Ai–Ci) exemplify the material morphologies in random sections after the thresholding performed in CTAn. Figure 9(Aii–Cvii) is captured in CTvox and illustrates the following: Figure 9(Aii–Cii)—square unit of the 3D printed object, Figure 9(Aiii–Ciii)—square unit with reconstructed pores within, Figure 9(Aiv–Civ)—cross section of the 3D printed object with reconstructed pores, Figure 9(Av–Cv)—extended view of 4 square units and filament junction, Figure 9(Avi–Cvi)—3D printed object with reconstructed pores in half its volume, Figure 9(Avii–Cvii)—close-up of one exterior round wall of the printed object. The color legend associated with the reconstructed pore domain is: violet → [6–102 μm), green → [102–198 μm), blue → [198–294 μm), pink → [294–390) and white → larger than 390 μm.

The morphologies of porous 3D printed specimens of 20%GelMA-3%HC5, 30%GelMA-3%HC5 and 35%GelMA-3%HC5 were investigated by means of µCT. Various angles of the three samples are illustrated in Figure 9 sections A–C divided for each composition. Overall, fair fidelity with the predesigned printing model can be observed for the three formulations processed by controlled extrusion followed by drying. Thicker configurations of the printed object occur where deposited filaments superimpose. Nonetheless, the internal architecture is rather homogeneous throughout the object volume. Subdivisions Figure 9(Ai–Ci) of the A–C sets depict 2D cross sections of 20%GelMA-3%HC5, 30%GelMA-3%HC5 and 35%GelMA-3%HC5 as recorded in CTAn software. The samples exhibit a dense structure and a wide array of interconnected pores. The scaffold support can provide stiff centri for osseous lineage adhesion and in-volume proliferation. Notably, the 2D section of all samples mirror an interesting cancellous bone-like morphology [34]. The microarchitercural pattern was deemed fitter for bone cell progenitors to proliferate within suchlike scaffolds with trabecular inspired frameworks [35].

As a whole, the printability of the three composite formulations differing with respect to the concentration of the organic matrix can be appraised via Figure 9(Aii–Cii) and Figure 9(Avii–Cvii) subsets; where distinct differences can be observed regarding the circular domains on the outskirts of the object versus the 90 degree angles of the inner pattern.

Detailed image refinement enabled the reconstruction of the void cavities inside the prints’ volume as independent objects. In CTVox, we superimposed the solid sample and diameter-restricted pore tomograms and illustrated them together. Thus, we aimed to assess the homogenous distribution of different sized pores in the scaffold volume (v) subsets of A–C assortments of Figure 9 provide a global view of the 20%GelMA-3%HC5, 30%GelMA-3%HC5 and 35%GelMA-3%HC5 tomograms and the even scattering of color-highlighted pores within the bounds of the prints. Despite the fact that upon drying some domains of the printed object became thinner to the benefit of the more solid content found at the filament cross sections the variety of pores of various size distributed is very good. This balanced distribution between the small and large pore domains is a superb trait of scaffolds with even mechanical support on the whole and durotactic gradients in macroscale. Coupled with the interconnectivity of the pores, this is an ideal feature which can support bone progenitors throughout all of the phenotypic differentiation stages. Detailed views of the pore spreading are featured in subdivisions Figure 9(Aiii–Ciii) and Figure 9(Aiv–Civ), in cross section.

Section D plots the quantitative values measured in CTAn for the solid features and pore domains. Apart from the charted data, we quantified the T.Po. as a percentage of the three 3D prints. In particular, the porosity shift versus GelMA concentration is as following: T.Po_20%GelMA-3%HC5_ > T.Po_30%GelMA-3%HC5_ > T.Po_35%GelMA-3%HC5_ (73.76 > 55.60 > 51.51%). With respect to pores, the histogram depicts the tendency of larger pores to form in a lesser extent as the organic content increases. 20%GelMA-3%HC5 and 30%GelMA-3%HC5 have a bell-shape distribution, quite even, with maximum incidence of 318 ± 24 μm and 126 ± 24 μm, respectively. For the highest GelMA concentration, however, a linear variation of pore domains emerges, whereby most (52%) of the pores achieve a maximum extent of 54 μm and for all the rest of the intervals the incidence drop is consistent. Conversely, wall solidity was found to vary linearly for the composites with 20, 30 and 35% GelMA. Most solid structures befall under 100 μm, indicating that a large share of the walls are thin. These kind of networks enveloping the pore palette must be consistent throughout the object since they span up to 58 %, 85% and 88% of all the solid layout of the prints, in an increasing order of GelMa concentration. Regarding the wall isotropy, the size field for 20%GelMA-3%HC5 seems to be the most limited due to the least solid content, with very little incidence above 200 μm width; the most uniform distribution emerges for 30%GelMA-3%HC5. 35%GelMA-3%HC5 exhibits an interesting behavior as for the highest GelMA amount, we expected thicker wall formation. Still, there is a very interesting balance between pore/wall dimensions, which vary remarkably in the same linear model, though with different ratios vis-à-vis the size domains and their total (more than 99.4% of all pores and walls are narrower than 300 μm).

#### 2.2.3. Swelling Degree, Degradability of the 3D Printed Hydrogel Based on GelMA

Swelling and degradation analyses are some of the most important parameters to be studied for hydrogels, which are intended to be used in tissue engineering, because they measure the hydrophilic or the hydrophobic character of the material. These parameters can be influenced by the methacrylation degree of GelMA, the amount of the photoinitiator used and time of UV curing. Furthermore, the hydrophilic/hydrophobic character of the material is certainly influenced by GelMA concentration [17,36,37].

**Figure 10:**
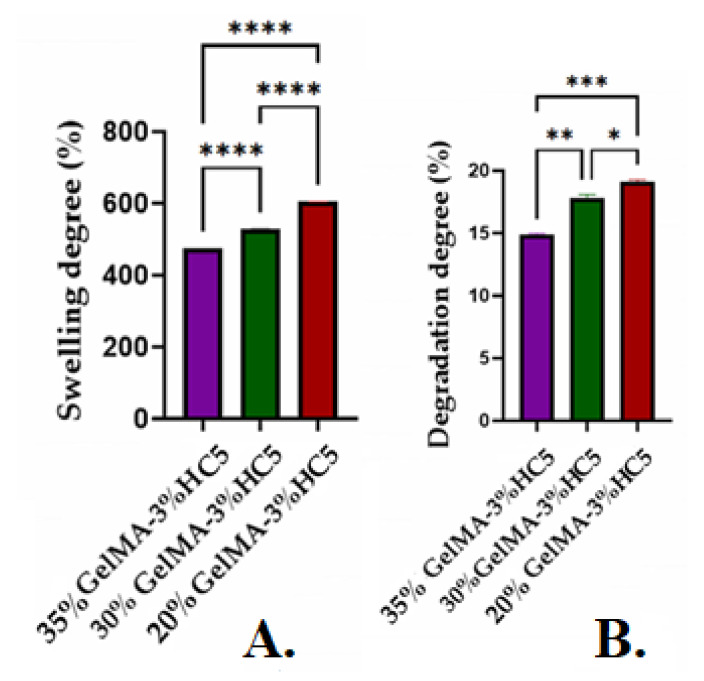
(**A**) Swelling degree determined on swollen samples at equilibrium, and (**B**) degradation degree determined at 24 h. Statistical significance: **** *p* < 0.0001, *** *p* < 0.001, ** *p* < 0.01, * *p* < 0.1.

As shown in Figure 10A, with increasing polymer concentration, the porosity of the scaffolds decreased, along with the degree of swelling. The decrease in swelling can also be supported by the hypothesis that with the increase in GelMA concentration, higher crosslinking density was obtained, explained by the denser internal architecture that generates a lower porosity, as shown in the micro-CT analyses (Figure 9) [36,37].

Degradation studies were also influenced by GelMA concentration. As shown in Figure 10B, the degradation rate decreased with an increase in GelMA concentration.

All these results are in good agreement with other research studies, which demonstrated that, as the GelMA concentration increased, lower degree of swelling and degradation were obtained [17,36,37].

#### 2.2.4. Nanoindentation

Nanoindentation analyses were performed on the photocrosslinked scaffolds. Before being analyzed, all samples were brought to the equilibrium swelling.

**Figure 11:**
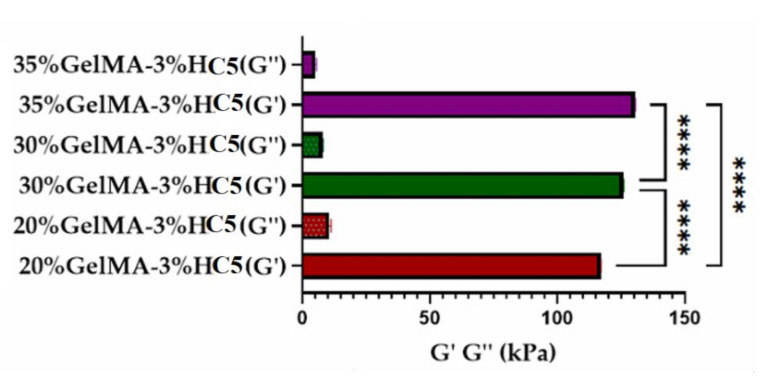
Storage and loss moduli determined by nanoindentation technique. Analyses performed on equilibrium swollen samples. Statistical significance: **** *p* < 0.0001.

All the nanocomposites’ scaffolds presented a storage modulus higher than loss modulus (G’ > G’’), i.e., a dominant elastic behavior rather than viscous character, these parameters suggest that all the samples behaved as crosslinked hydrogels (Figure 11).

Additionally, the mechanical analyses pointed out that as the polymer concentration increases, the degree of crosslinking increases. This behavior was explained by the decrease in porosity as the polymer concentration increased. These results are in good agreement with CT, swelling and degradation analyses.

#### 2.2.5. Evaluation of Cellular Response toward 3D Printed Scaffolds

**Figure 12:**
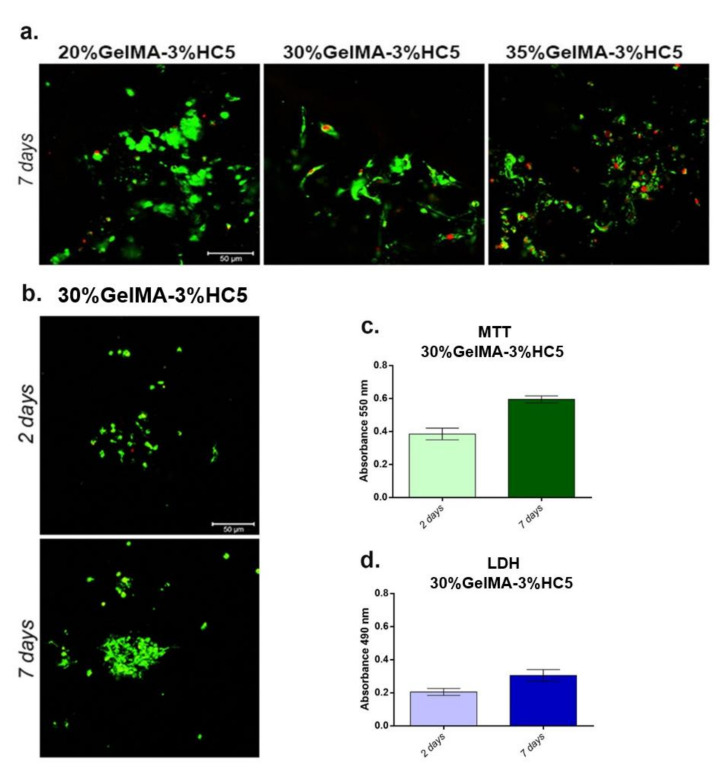
(**a**) Qualitative Live/Dead staining, showing live cells (green fluorescence) and dead cell nuclei (red fluorescence) in contact with 20%GelMA-3%Hap-Cerium, 30%GelMA-3%Hap-Cerium and 35%GelMA-3%Hap-Cerium. Scale bar 50 µm. (**b**) Fluorescence microscopy evaluation of living cells (green) and dead cells (red) in contact with GelMA-HC5 printed scaffold. Scale bar 50 µm. (**c**) Cell viability and proliferation profile evaluation by MTT assay at two and seven days after cells contact with 30%GelMA-3%HC5 scaffold. (**d**) 30%GelMA-3%HC5 scaffold cytotoxicity evaluation by LDH assay at two and seven days after cell seeding.

According to the Live/Dead results (Figure 12a), the addition of GelMA to 20% of the scaffold structure favored the formation of additional large-sized pores and determined the formation of cell groups preventing the achievement of an elongated cellular phenotype. On the other hand, the addition of GelMA to 30% of the scaffold structure determined the formation of medium-sized pores, which encouraged cell adhesion and viability, unlike 35% GelMA-based material, which formed smaller pores and produced a limiting effect on cell proliferation.

On the other hand, the biocompatibility assessment of the printed 30%GelMA-3%HC5 scaffold was performed after two and seven days after cell seeding. Live/Dead fluorescent staining and confocal microscopy were used to observe the ratio between live and dead cells in 3D systems after two and seven days of culture in standard conditions. The results indicated a good cell viability rate, probably due to the natural compounds in the biomaterials’ structure. Additionally, cell viability, proliferation and grouping were higher after seven days of culture (Figure 12b). The rate of viability and proliferation of MC3T3-E1 cells put in contact with 30%GelMA-3%HC5 printed scaffold was evaluated by MTT assay at two and seven days after seeding. The results indicated that MC3T3-E1 cells in contact with 30%GelMA-3%HC5 showed a high viability profile after two days of culture, indicating that the combination between GelMA and 5% Cerium provide a proper environment for cells. From two to seven days of culture, cell viability increased, suggesting cell growth and proliferation in contact with 30%GelMA-3%HC5 (Figure 12c). The cytotoxicity of the printed scaffold was evaluated using LDH assay and a low cytotoxicity profile was registered after two and seven days of culture. These results suggest that the combination between GelMA and 5% Cerium and 3D printing process promote a favorable cellular response (Figure 12d).

#### 2.2.6. Evaluation of 30%GelMA-3%HC5 Printed Scaffold Effect on Osteogenic Differentiation

In order to examine the osteogenic differentiation of MC3T3-E1 in contact with 30%GelMA-3%HC5 scaffold, we analyzed the expressions of early and late osteogenic markers by immunofluorescence and qPCR, at 14 and 28 days after induction.

**Figure 13:**
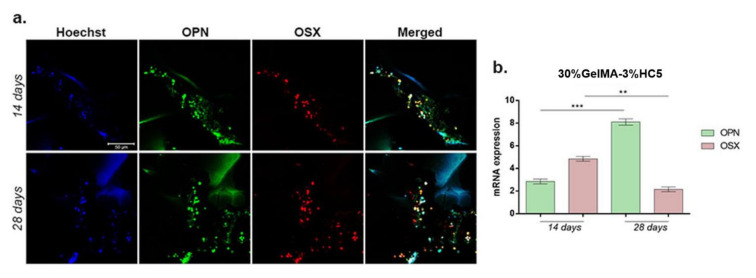
(**a**) OPN and OSX protein expression visualized by immunofluorescence coupled with confocal microscopy after 14 and 28 days of osteogenic differentiation in contact with 30%GelMA-3%HC5 scaffolds. Cells nuclei are stained with Hoechst, OPN is stained in green (FITC) and OSX is stained with red (TRITC). Scale bar 50 µm. (**b**) OPN and OSX gene expression after 14 and 28 days of osteogenesis in contact with 30%GelMA-3%HC5 scaffolds. Statistical significance: ** *p* < 0.01, *** *p* < 0.001.

OPN is a late osteogenic marker specific for bone matrix, while OSX is an early osteogenic marker. Immunofluorescence targeting OPN and OSX was performed. OPN was expressed in the bone matrix and was more intense after 28 days of osteogenesis. OSX resulted in a strong staining, although a more intense staining was found after 14 days of osteogenesis (Figure 13a). Gene expression studies revealed a statistically significant increase in OPN gene expression after 28 days of osteogenesis (*p* < 0.001) compared to 14 days in contact with 30%GelMA-3%HC5 scaffold (Figure 13b). Furthermore, the results displayed OSX activation and an increased expression profile at 14 days after induction of osteogenesis. Once the process was induced, OSX levels significantly decreased up to 28 days (*p* < 0.01). This profile is typical for a gene with early activation in osteogenic differentiation process and confirms the ability of 30%GelMA-3%HC5 scaffold to support and efficiently maintain osteogenesis.

## 3. Materials and Methods

### 3.1. Materials

Ceramic powders of hydroxyapatite doped with cerium ions were synthesized in our laboratory, by the method of precipitation from aqueous solutions (co-precipitation reactions), using the following raw materials: calcium nitrate (Ca(NO_3_)_2_•4H_2_O ≥ 99.0%), dibasic ammonium phosphate ((NH_4_)_2_HPO_4_ ≥ 98%), ammonium hydroxide solution (NH_4_OH, 28.0–30.0% NH_3_), cerium (III) nitrate (Ce(NO_3_)_2_•6H_2_O). All raw materials was purchased from Sigma Aldrich, Eschenstrasse 5 Taufkirchen, D-82024, Germany.

Dulbecco Modified Eagle’s Medium (DMEM) low glucose, antibiotic antimycotic solution, phosphate buffered saline (PBS) powder, 3-(4,5-dimethylthiazolyl-2)-2,5-diphenyltetrazolium bromide (MTT) reagent, “In vitro toxicology assay kit lactate dehydrogenase (LDH) based” (TOX7-1KT) assay kit, Triton-X100, paraformaldehyde (PFA) solution and Hoechst 33258 solution for fluorescence staining of cell nuclei were purchased from Sigma-Aldrich, Germany. Fetal bovine serum (FBS) and Live/Dead assay kit were purchased from Thermo Fisher Scientific, Waltham, MA, USA. TRIzol Reagent, goat anti-mouse secondary antibody AlexaFluor 488 (A11029), and goat anti-rabbit secondary antibody AlexaFluor 546 (A11010) were purchased from Invitrogen, USA. Osteopontin (OPN) (sc-21742) and osterix (OSX) antibodies (sc-393325) were purchased from Santa Cruz Biotechnology, Inc. iScript cDNA Synthesis kit was purchased from BioRad, 1000 Alfred Nobel Drive Hercules, California 94547, USA.

The necessary quantities of raw materials were determined by performing calculations to obtain Ca_10-x_Ce_x_(PO_4_)_6_(OH)_2_, xCe = 0.1, 0.3, 0.5, with respect to the molar ratio (Ca + Ce)/P of 1.67. The samples name and the doping concentrations are presented in Table 1.

Gelatin Methacryloyl (GelMA) was synthetized using: Gelatine type B, from bovine skin (Sigma-Aldrich, St. Louis, MO, USA); methacrylic anhydride (MA) (Sigma-Aldrich, Goettingen, Germany) and 2-hydroxy-40-(2hydroxyethoxy)-2-methylpropiophenone Irgacure 2959 (I-2959) (Sigma-Aldrich, Milano, Italy). The raw materials were used as received. 

### 3.2. Synthesis of Ceramic Powders

**Figure 14:**
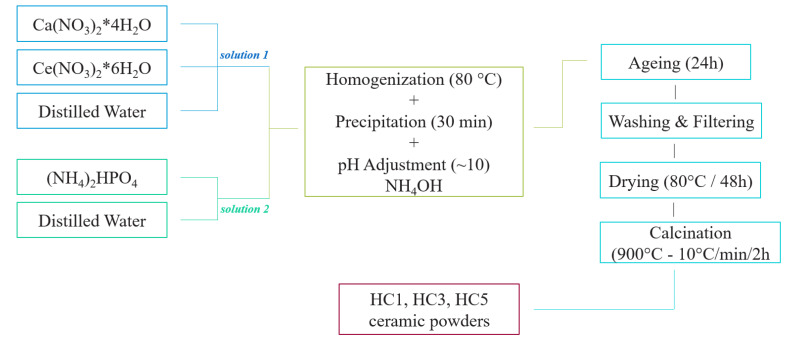
Schematic representation of obtaining by co-precipitation reaction of hydroxyapatite powders doped with cerium ions—HC1, HC3 and HC5.

In order to obtain ceramic hydroxyapatite powders (HAp) doped with cerium ions, the wet synthesis route was used by co-precipitation method (Figure 14).

The precursors were homogenized in distilled water for about 30 min and two solutions were obtained - solution 1, consisting of calcium nitrate and cerium nitrate, and solution 2 consisting of dibasic ammonium phosphate. Subsequently, the homogenization process took place by adding solution 2 drop wise over solution 1 at a temperature of 80 °C, in order to obtain a milky white precipitate.

After obtaining the precipitate, the pH of the medium was checked and adjusted to a value of about 10, with a solution of ammonium hydroxide and then left to mature at room temperature for two days. Then, the matured precipitate was washed with distilled water several times and was subjected to the drying process at a temperature of 80 °C for about 48 h.

The dry precipitate was characterized by complex thermal analysis (DTA-TG) and X-ray diffraction (XRD) in terms of phase composition. The last step in the process of obtaining HAp doped with cerium ions, was to thermally treat (calcination) the dry precipitate, at a temperature of 900 °C, with a speed of 10 °C/min and with a threshold of 2 h. Thus, the ceramic powder was subsequently characterized for phase composition by X-ray diffraction, morphological composition by Brunauer-Emmett-Teller (BET) and dispersion characteristics and grain size distribution by laser diffraction analysis.

### 3.3. Synthesis of GelMA

Initially, gelatin was dissolved in PBS (pH 7.4) at 50 °C. After the solubilization of gelatin (~1 h), methacrylic anhydride was added drop by drop and allowed to react with the functional groups of gelatin for 2 h, under magnetic stirring at 50 °C. Finally, the unreacted methacrylic anhydride was removed by dialysis at 40 °C over 5 days, using cellulose dialysis bags and distilled water. The obtained GelMA was dried by lyophilization at 0.025 bars for 24 h and stored at 4 °C in dry conditions.

### 3.4. Synthesis of Composite Materials

The second part of this study followed the investigation and selection of the optimal concentration of HAp doped with cerium ions (C1, C3, C5), which will ensure GelMA-based scaffolds with good biocompatibility, viability and cell proliferation.

Composites materials based on GelMA-HC1, GelMA-HC3, GelMA-HC5 were obtained as follows: Briefly, hydroxyapatite doped with cerium ions (3% (w/v%) from the total amount of GelMA) was subjected to dispersion in PBS, for 1 h and sonicated for 5 min. Then, GelMA was added at a concentration of 20% (w/v%) and the resulted system was left for 24 h at 40 °C. Finally, the photoinitiator was added in a concentration of 1% from the total amount of polymer (w/v%). When the photoinitiator was completely dissolved, the material was inserted into the 3D printing cartridge and allowed to reach room temperature before printing.

Based on the biological results obtained in the first part of the study, a fixed concentration of hydroxyapatite doped with cerium ions was selected (sample HC5) to be used in the next part of the study.

Therefore, the third part of this study aimed to select the optimal concentrations of GelMA for the 3D printing process.

To enrich the nanocomposite hydrogel-based printing inks, three concentrations of GelMA were selected: 20%, 30% and 35% (w/v%). Then, the above-described working protocol (which was used in the second part of the study) was applied to obtain the composite materials with different amounts of GelMA and a constant HC5 concentration of 3% (w/v%) as follows: HC5 in a concentration of 3% (w/v%) from the total amount of GelMA was subjected to dispersion in PBS for 1 h and sonication for 5 min. Then, GelMA was added at a concentration of 20%/30%/35% (w/v%) and left to react for 24 h at 40 °C. Finally, the photoinitiator was added in a concentration of 1% (w/v%) from the entire quantity of GelMA and was subjected to homogenization. When the photoinitiator was completely dissolved, the material was inserted into the 3D printing cartridge and allowed to reach room temperature before the printing stage.

### 3.5. Characterization Techniques of Ceramic Powders, GelMA and Composite Materials

#### 3.5.1. X-ray Diffraction (XRD)

The composition and crystallinity of dry precipitates and final ceramic powders were evaluated by X-ray diffraction (XRD), performed with a Shimadzu XRD 6000 diffractometer (Shimadzu, Kyoto, Japan), with Ni filtered CuK α radiation (α = 1.5406 Å), 2 theta in the range 20–70°, with a scan step of 0.02° and a counting time of 0.6 s/step. The average diameter of crystallite, D (nm) was calculated using the Scherrer equation (Equation (1)):

(1) $$D=\frac{K\lambda }{\beta \cos\theta }$$

where D is the average particle diameter (Å), K is a constant (0.94), λ is the X-ray radiation wavelength (1.5406 Å), θ is the peak angle and β is the width at half maximum (FHWM) of the respective XRD peak [38].

#### 3.5.2. Brunauer–Emmett–Teller (BET)

The specific surface area and the pore size dimension were assessed by the Brunauer–Emmett–Teller (BET) analysis, which was performed on a Micrometrics Gemini V2 model 2380 (Micromeritics Instruments Corporation, Norcross, GA, USA). The adsorption isotherms were obtained by measuring the amount of gas adsorbed across a wide range of relative pressures at a constant temperature (N2, 77 K and pressure between 780 and 7.8 mmHg). Conversely desorption isotherms were achieved by measuring the gas removed as pressure was reduced.

#### 3.5.3. Laser Diffraction Granulometer

The particle size distribution of ceramic powders was evaluated using the Malvern Mastersizer 2000 laser diffraction granulometer (Malvern Instruments, Malvern, UK).

#### 3.5.4. Scanning Electron Microscopy SEM

The morphology was assessed by scanning electron microscopy (SEM) using a Quanta Inspect F50 FEG scanning electron microscope, with a resolution of 1.2 nm (Thermo Fisher, Eindhoven, The Netherlands); the composite materials were covered with a thin gold layer [39].

#### 3.5.5. ^1^H-NMR Spectrometry

The methacrylation degree of GelMA was determined by ^1^H-NMR spectrometry. Analyses were performed on Bruker NMR 600 MHz Advance spectrometer (Bruker BioSpin Rheinstetten, Germany), using 20 mg GelMA dissolved in 0.70 mL D_2_O.

Using Equation (2) the methacrylation degree was calculated.

(2) $$\mathrm{Methacrylation}\;\mathrm{degree}\;(\%)=1-\frac{\mathrm{integration}\;\mathrm{signal}\;\mathrm{of}\;\mathrm{arginine}\;\mathrm{from}\;\mathrm{GelMA}\;}{\mathrm{integration}\;\mathrm{signal}\;\mathrm{of}\;\mathrm{arginine}\;\mathrm{from}\;\mathrm{Gelatin}}\;\times \;100$$

#### 3.5.6. FTIR Spectrometry

FTIR spectra were recorded on a Bruker VERTEX 70 instrument, using the total reflection attenuation module (ATR) at a resolution of 4 cm^−1^, range of 600–4000 cm^−1^ and the final spectra represent the average of 32 scans.

#### 3.5.7. Biological Analyses

Before performing all the assays, the materials were sterilized using UV light and cut in 1 cm^2^ diameter pieces. MC3T3-E1 cells were seeded on the surface of the porous materials at 2 × 10^5^ cells/cm^2^ density, allowed to populate the entire structure of the 3D scaffolds and incubated in standard conditions until the biocompatibility assays were performed.

- Cell culture model. Murine preosteoblasts from MC3T3-E1 cell line (ATCC CRL-2593) were used for the biocompatibility assessment. MC3T3-E1 cells were cultured in DMEM media supplemented with 10% FBS and 1% antibiotic antimycotic solution and maintained at 37 °C, 5% CO_2_, in a humidified atmosphere.
- Biocompatibility assays. To evaluate the biocompatibility of the GelMA-Hap enriched 3D scaffolds, cells’ viability, proliferation profile and materials’ cytotoxicity were evaluated at two and seven days after cells seeding. Cell viability and proliferation profile were quantitatively determined by MTT assay and qualitatively by Live/Dead fluorescence staining, while the materials’ cytotoxicity was quantitatively assessed by LDH assay.
- MTT assay. Cells’ viability and proliferation profile were qualitatively determined using spectrophotometric MTT assay, at two and seven days after seeding. The 3D systems were incubated with 1 mg/mL MTT solution prepared in simple culture media, for 4 h at 37 °C, allowing the formation of formazan crystals by living cells. The formazan crystals were solubilized with isopropanol and the optic density was measured by spectrophotometry at 550 nm on Flex Station 3 (Molecular Devices, LLC. 3860 N First Street San Jose, CA 95134, USA)). The values obtained were directly proportional with the amount of live cells.
- LDH assay. The cytotoxic potential of GelMA-HAp enriched 3D scaffolds was determined based on LDH quantification, released from damaged cells, which lost their membrane integrity. The culture medium was collected from the 3D systems and mixed with LDH Assay Kit reagents, according to the manufacturer’s instructions. The mix was stored in the dark for 10–15 min and the optic density was measured by spectrophotometry at 490 nm on Flex Station 3. The quantity of LDH was directly proportional with the number of dead cells.
- Live/Dead fluorescence microscopy. The ratio between live cells and dead cells was qualitatively evaluated via Live/Dead fluorescence staining. The assay provided two fluorescent dyes, calcein acetomethoxy (AM), which marks live cells in green fluorescence, and ethidium bromide homodimer (EtBr), which marks dead cells in red fluorescence. The staining solution was prepared according to the manufacturer’s indications and incubated with the 3D systems for 1 h at room temperature, in the dark. The images were captured using confocal microscope Zeiss 710 and processed using Zeiss Zen software.
- Statistical analysis. All experiments were performed in triplicate (n = 3). Statistical analysis of the data was performed using GraphPad Prism software, one-way ANOVA method and Bonferroni algorithm to compare between groups. Values were considered significant for *p* < 0.05.

### 3.6. Characterization of 3D Scaffolds Obtained from Nanocomposite Hydrogel Printing Inks—20% GelMA-3% HC, 30% GelMA-3% HC, 35% GelMA-3% HC

#### 3.6.1. Printability

In order to explore the printability of the nanocomposites-based inks and to obtain GelMA-based 3D structures, the 3D Discovery ™ V6.1 bioprinter (RegenHU, ZI du Vivier 22, 1690Villaz-St-Pierre, Switzerland) was employed.

Three-dimensional printing involves making a three-dimensional object by the printing inks as superimposed layers. To obtain objects of different shapes and sizes, the printer is equipped with software for 2D/3D design.

In this study, BIOCAD software was selected and, out of the four print heads with which the printer is equipped (coaxial, direct dispensing, melt electrospinning and cell friendly), the direct dispensing print head of the 3Dbioprinter was used. Additionally, to establish the 3D printing parameters, printing speeds in the range of 2–10 mm/s, pressures with values of 90 to 310 kPa and cylindrical nozzles of 23G, 25G were tested. Scaffolds were printed at room temperature and UV cured at 365 nm.

#### 3.6.2. Swelling Degree, Degradability of the 3D Printed Scaffolds

Swelling and degradability analyses were performed in triplicate, using the 3D printed samples. After the printing process, the samples were lyophilized, weighed, and then introduced in PBS. For swelling analyses, samples were weighed after predetermined periods of time. The maximum degree of swelling was considered at the time when the samples reached constant mass after two weight determinations.

For degradability analyses of the 3D printed samples, scaffolds were introduced in PBS for 24 h, then dried by lyophilization and weighed.

Swelling degree was determined using Equation (3) and dissolvability using Equation (4).

(3) $$\mathrm{Swelling}\;\mathrm{degree}\;(\%)=\frac{\mathrm{Ww}-\mathrm{Wd}}{\mathrm{Wd}}\times 100$$

where Ww = wet weight, Wd = dry weight

(4) $$\mathrm{Degradation}\;\mathrm{degree}\;(\%)=\frac{W0-\mathrm{Wd}}{\mathrm{Wd}}\times 100$$

where Wo = initial weight, Wd = dry weight

#### 3.6.3. Microcomputer Tomography (µCT)

The high-resolution Bruker CT 1272 equipment was employed for the micro-computer tomography analysis. The scanning was performed without a filter, with a 50 kV source voltage, a 130 μA current intensity, and a 450 ms exposure time each frame. The scanning was conducted while the samples were rotated 180° with a 0.2° rotation step. The image was created by averaging three frame acquisitions for each unique slice. The picture pixel size for each of the three samples was set to 6 μm.

In Bruker NRecon 1.7.1.6 software, tomograms were reconstructed from raw data (Kontich, Belgium). Beam hardening correction was set to 7–9, ring artefact reduction was set to 5, and smoothing was set to 1. CTVox (Bruker) was used to visualize reconstructed tomograms, and CTAn 1.17.7.2 was used to do sample analysis (Bruker, Kontich, Belgium). One volume-of-interest (VOI) dataset was extracted for each material, consisting of 100% of the scanned object and limited to its exterior surfaces.

The VOIs were subjected to an image-processing task list in CTAn that included thresholding (to singularly separate the specimen walls from its pores), despeckling (to remove residual scanning artefacts) and 3D analysis for the numerical quantification of total porosity (T.Po.) “structure separation” (pore dimensions) and “structure thickness” (wall thickness). After thresholding, the tomogram pixels were binarized (all the pixels associated with the solid sample were converted in white while the rest were black, depicting the pores within the sample and also the environment outside of it).

Based on the scanning resolution of 6 μm, quantitative analysis was performed by computing the object feature size equivalent to respective white/black 3D pixels to determine the width domains of the solid prints/specific porosity. The amassed values are delivered on fixed intervals, starting with the scanning resolution up to threefold its value and so on (e.g., 6–18 μm, 18–30 μm, 30–42 μm, 42–54 μm, etc). For convenience, the associated values of these domains can be grouped upon reporting.

In addition, CTAn tool enables the separation of size-specific object features, in particular specific pore domains. In order to do this, after binarizing the dataset, the images were inverted; this process enabled the visualization and measurement of the pores as solid objects and their separation based on desired boundaries. For 20% GelMA-3% HAP-Cerium, 30% GelMA-3% HAP-Cerium, 35% GelMA-3% HAP-Cerium, we performed this process in order to extract the 3D tomograms of the pores on the following intervals: 6–102 μm, 102–198 μm, 198–294 μm, 294–390 and larger than 390 μm. CTVox was used to visualize the pore tomograms, loaded within the 3D solid print, to better comprehend the interface and distribution of pore network.

#### 3.6.4. Nanoindentation

Nanoindentation tests were executed using Nano Indenter^®®^ G200 (Santa Clara, CA, USA). Tests were performed in triplicate using G-Series DCM CSM Flat Punch Complex Modulus Gel. Using this method, the storage modulus G’ and loss modulus G’’ were determined at a predefined frequency.

#### 3.6.5. Evaluation of Osteogenic Markers Gene and Protein Expression

OPN and OSX gene expression was analyzed by qPCR. The total RNA was isolated from the obtained 3D systems using TRIzol Reagent according to the manufacturer’s indications. Total RNA was tested for purity and concentration using NanoDrop spectrophotometer (Thermo Fisher Scientific, Waltham, MA, USA) and an RNA integrity number (RIN) was determined using Agilent 2100 BioAnalyzer (Agilent Technologies, Hewlett-Packard-Strasse 8 76337 Waldbronn, Germany). Further, total cellular RNA was reverse-transcribed to complementary DNA (cDNA) using iScript cDNA Synthesis kit (BioRad 1000 Alfred Nobel Drive Hercules, California 94547, USA). The gene expressions of OPN and OSX was evaluated by qPCR, using SYBR Green method and ViiA7 equipment (Thermo Fisher Scientific, Waltham, MA, USA). Every sample was evaluated in triplicate, and the expression of GAPDH was used as a reference gene.

OPN and OSX protein expression was investigated by immunostaining and confocal microscopy. Bioconstructs were fixed with a 4% PFA solution for 1h and permeabilized with 0.1% Triton X100 solution in 2% BSA for 20 min, at 4 °C. Next, the 3D systems were incubated overnight with mouse monoclonal OPN and rabbit polyclonal OSX and then with goat anti-mouse secondary antibody AlexaFluor 488, and, respectively, goat anti-rabbit secondary antibody AlexaFluor 546, for 1 h at 4 °C. Cell nuclei were stained with Hoechst 33258 solution. All the images were visualized and captured using confocal microscope Zeiss 710 and processed using Zeiss Zen software.

## 4. Conclusions

This work reported the development and investigation of composite materials based on GelMA and HAp doped with cerium ions, as efficient biomaterials with potential applications in bone regeneration. The XRD analysis confirmed the modification of HAP with cerium ions, while the structural investigations (1H-NMR and FTIR) validated the successful grafting of methacrylic functionalities on the gelatin chains, respectively, the synthesis of GelMA. The SEM morphological analysis showed the formulation of composite materials with high interconnected porosity and fine distribution of mineral particles on the surface as well as within the polymer matrix, following the dispersion of HC5. The in vitro biological assessment presented the highest level of cell viability and proliferation potency (MTT test) of GelMA-HC5 composites along with a low cytotoxic potential (LDH assay), highlighting the beneficial effects of cerium on cell growth, which was also supported by Live/Dead results.

Further, according to the 3D printing experiments, among the tested GelMA concentrations (20%, 30%, 35%), the inks based on 30% GelMA enriched with HC5 were capable of generating 3D scaffolds with high structural integrity and homogeneity, showing the highest suitability for the 3D printing process. Even if micro-mechanical and degradation investigations showed the superiority of 35% GelMA-3%HC5 in terms of micromechanical properties and stability, originated from the presence of a denser internal architecture that generates a lower porosity (confirmed in micro-CT analyses), these materials presented the lowest cellular response, limiting the cell proliferation (Live/Dead assay). On the other side, the highest biocompatibility and ability to promote and support the proliferation of MC3T3-E1 cells culture, registered in the case of 3D printed scaffolds based on 30% GelMA-3%HC5 incubated with the cell culture, was corelated with their high porosity.

After selecting the ideal composition, 30 GelMA-3%HC5 3D printed scaffolds were investigated to evaluate their ability to be used in tissue engineering, thus examining the osteogenic differentiation of MC3T3-E1 in contact with the mentioned structures, analyzing the expressions of osteogenic markers. Gene expression studies have shown a statistically significant increase in OPN gene expression after 28 days of osteogenesis. Furthermore, the results showed OSX activation and an increased expression profile at 14 days after osteogenesis induction. Once the process was induced, OSX levels dropped significantly to 28 days. This profile is typical of a gene with early activation in the process of osteogenic differentiation and confirms the ability of 3D printed structures to effectively support and maintain osteogenesis.

Hence, based on the obtained results, 30 GelMA-3%HC5 3D printed scaffolds could be considered as biomaterials with suitable characteristics for application in bone tissue engineering. Additionally, the 30 GelMA-3%HC5 3D printed scaffolds, with high-performance properties and the capacity for osteogenic differentiation, can have a significant impact on people’s quality of life, providing a way to easily heal bone defects.

## Figures and Tables

**Table 1:** Doping concentrations of cerium ions for hydroxyapatite ceramic powders.

*Sample*	*Cerium Ion Concentration (Molar %)*
*HC1*	0.1
*HC3*	0.3
*HC5*	0.5

## Data Availability

Not applicable.
